# Comparability of 24-hour composite and grab samples for detection of SARS-2-CoV RNA in wastewater

**DOI:** 10.1093/femsmc/xtac017

**Published:** 2022-06-17

**Authors:** Brittany L Kmush, David Monk, Hyatt Green, Darcy A Sachs†, Teng Zeng, David A Larsen

**Affiliations:** Department of Public Health, Syracuse University, Suite 444 White Hall, Syracuse, NY 13244, United States; Arcadis, 110 W Fayette St, Syracuse, NY 13202, United States; Department of Environmental Biology, State University of New York College of Environmental Science and Forestry, 1 Forestry Dr., Syracuse, NY 13210, United States; Arcadis, 110 W Fayette St, Syracuse, NY 13202, United States; Department of Civil and Environmental Engineering, Syracuse University, 151 Link Hall, Syracuse, NY 13244, United States; Department of Public Health, Syracuse University, Suite 444 White Hall, Syracuse, NY 13244, United States

**Keywords:** SARS-CoV-2, wastewater, grab samples, composite samples, infectious disease, surveillance

## Abstract

Wastewater surveillance is a cost-effective way to monitor pathogen prevalence and transmission patterns in the entire community. Here, we compare 24-hour composite and grab samples collected during September 2020 from several municipalities in New York State to detect SARS-CoV-2. A total of 45 paired samples (90 total samples) from three counties and 14 wastewater treatment plants were available for analysis. The categorical comparison (SARS-CoV-2 genetic material detected and quantifiable, genetic material detected but below the limits of quantification, and genetic material not detected) between the grab and composite samples was quite strong, with 91.1% agreement (kappa *P*-value < .001). The correlations among the quantifiable grab and composite samples were statistically significant yet modest for SARS2-CoV RNA (Pearson correlation = 0.44, *P* = .02), crAssphage cDNA (Pearson correlation = 0.36, *P* = .02), and crAssphage DNA (Pearson correlation = 0.46, *P* = .002). We found good comparison between grab and 24-hour composite samples for detecting SARS-CoV-2 RNA from municipal wastewater treatment plants. Grab sampling is an efficient and cost-effective method to monitor for the presence of SARS-CoV-2 in the entire community.

## Teaser

Wastewater grab samples are efficient and compare well to 24-hour composite samples when monitoring for the presence of SARS-CoV-2 in a low transmission community.

## Introduction

Wastewater surveillance for infectious diseases has been used for decades, first gaining popularity during polio eradication campaigns (Vaccines and Biologicals World Health Organization 2003). Wastewater surveillance is a cost-effective way to monitor pathogen prevalence and transmission patterns in the entire community (Larsen and Wigginton 2020). With the start of the COVID-19 pandemic, several groups, including ours, were able to demonstrate the feasibility of detecting SARS-CoV-2 RNA in wastewater (Medema et al. 2020, Peccia et al. 2020, Wurtzer et al. 2020, Wilder et al. 2021). Soon after, municipalities across the globe decided to use the science to monitor SARS-CoV-2 transmission in their communities (Naughton et al. 2021). However, this unprecedented interest caused availability shortages in wastewater sampling equipment.

Generally, continuous flow-proportional sampling, where a small sample of wastewater is collected at a defined volume interval, is considered the gold standard. However, this is not always possible due to the inability to install flow-measuring equipment at sampling locations. Therefore, 24-composite samples, where a small sample of wastewater is collected at regular time intervals (such as every 15 minutes) for 24 hours and then combined together are often used as an industry standard (Schaeffer et al. 1980, Brumelle et al. 1984, Cornman et al. 2018, Water Research Foundation T 2020). This sampling method can account for variations in wastewater characteristics from the population it is monitoring and increase the validity of the sample. However, the autosamplers required to collect this type of sample are expensive, subject to availability issues, and cannot be used at all collection points. When an autosampler is not available, a one-time grab sample is often used instead. This method, however, is highly subjected to wastewater discharge patterns and the timing of the grab sample should be chosen strategically. Water soluble pharmaceuticals, personal care products, and illicit drugs are particularly susceptible to inadequate sampling strategies, and it is difficult to determine if variations in results are due to real changes or due to the sampling method (Ort et al. 2010). The SARS-CoV-2 is a solids-associated RNA virus; we aimed to assess the utility of grab samples as a tool to detect the virus.

During the summer of 2020, New York State implemented a pilot program in several municipalities to monitor SARS-CoV-2 transmission in wastewater. During the course of this program, we collected both a 24-hour composite and a grab sample from several municipalities. Here, we describe the comparability of these methods for detection of SARS-CoV-2 and crAssphage, a human fecal indicator (Stachler et al. 2017).

## Materials and methods

### Study design

The New York State Department of Health commissioned a pilot study of wastewater surveillance for SARS-CoV-2 in August of 2020. As many different SARS-CoV-2 analytical procedures have been developed by a host of public and private entities, we designed a study to compare results from 24-hour composite samples to well-timed grab samples, collected during the morning diurnal peak. This comparison was part of a greater quality assessment program to determine sampling and analytical consistency, repeatability, effective range, and limit of detection.

### Grab and 24-hour composite sample collection

Both grab and 24-hour composite samples were collected from specified locations in the cities of Albany, Buffalo, and Newburg in New York State as shown in Table 1. Autosamplers were set to collect at a frequency and sampling volume within parameters set forth by the US Environmental Protection Agency. A representative sample was collected from the 24-hour composite sampler, the samples were iced or refrigerated during the collection process. A grab sample was obtained at the same location and collected at the end of the 24-hour collection period. Both samples were transported in a cooler with ice to maintain a 4°C temperature. Both samples arrived at the analytical laboratory within 24 hours of collection.

**Figure 1.**
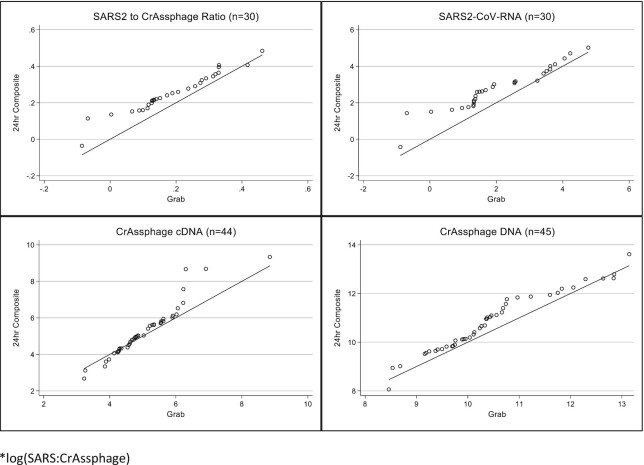
Quantile–quantile plots comparing grab and 24-hour composite samples (log values) *log(SARS: CrAssphage).

**Figure 2.**
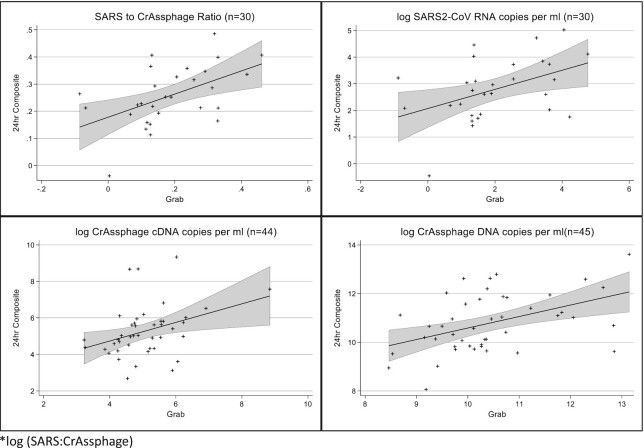
Correlations between grab and 24-hour composite samples (log values) *log (SARS: CrAssphage).

**Table 1.** Basic characteristics for grab and composite sampling locations in New York State, 2020.

Wastewater treatment plant	Sampling point	Number of paired samples	Description	Population*	Mean daily COVID cases during September 2020	Mean daily COVID cases during September 2020 per 100 000 population
Albany						
	AL-02	1	Suburban neighborhoods	3861	0.10	2.59
	AL-05	2	City and student neighborhood	9868	0.38	3.85
	AL-06	4	City neighborhood	7054	0.00	0.00
	AL-07	4	City neighborhood, outer downtown	2219	0.04	1.80
	AL-08	2	Downtown	2253	0.11	4.88
	AL-09	2	City neighborhood	3665	0.00	0.00
	AL-10	9	City neighborhood	3835	0.00	0.00
	AL-11	6	Municipal wastewater treatment plant	61 900	3.97	6.41
Buffalo						
	BU-01	2	South Buffalo and outside areas	67 271	4.47	6.64
	BU-07	1	City neighborhood	8641	0.69	7.99
	BU-08	1	City neighborhood, college housing	15 610	1.21	7.75
	BU-09	2	City neighborhood	6967	0.97	13.92
	BU-10	2	City neighborhood	4099	0.45	10.98
Newburgh						
	NE-01	7	Municipal wastewater treatment plant	54 000	2.14	3.96

*Estimated total.

Sample collection followed standardized operating procedures (SOPs) for sampling and a quality assurance/quality control (QA/QC) plan for all routine and point monitoring sampling. This plan included the use of field blank samples, blind duplicate samples, and matrix spike duplicate samples. The use of the SOPs minimized the variability associated with sampling and sample splitting in order to evaluate the inherent variability associated with wastewater, as well as the laboratory variability.

### SARS-2-CoV RNA and crAssphage DNA and RNA quantification

SARS-CoV-2 RNA and crAssphage DNA and cDNA were quantified as previously described (Wilder et al. 2021). In brief, samples were processed using the ultracentrifugation through a sucrose cushion technique before nucleic acid extraction and PCR quantification. This method has a limit of quantification of five copies per ml (Wilder et al. 2021). All wastewater samples were analyzed in triplicate.

### Case data collection

In order to confirm that cases were present in the sampled sewer sheds, we matched COVID-19 diagnosed cases and diagnostic tests from the New York State Electronic Clinical Laboratory System (ECLRS; New York State Department of Health 2021) to sewershed polygons generated in consultation with municipal sanitation engineers using the home address of each COVID-19 case.

### Statistical analysis

Each wastewater sample could have one of the three possible SARS-CoV-2 qualitative outcomes: genetic material detected and quantifiable, genetic material detected but below the limits of quantification, or genetic material not detected. The % agreement and kappa statistic were calculated for the categorical outcomes of the paired samples. The amount of genetic material in the quantifiable samples were log transformed. Among the log transformed quantifiable samples, the correlations between the grab and composite samples were plotted, pairwise Pearson correlation coefficients were calculated, and quantile–quantile plots were generated. Equality of variances between the grab and composite samples were compared using Levene's Robust Test statistic. We assessed four separate measures: log-transformed SARS-CoV-2, log-transformed SARS-CoV-2 normalized by dividing log-transformed crAssphage DNA, log-transformed crAssphage DNA, and log-transformed crAssphage cDNA. Two-sided *P*-values less than .05 were considered significant. These analyses were completed in Stata version 16.0.

## Results

A total of 45 paired samples (90 total samples) from three wastewater treatment plant sewer sheds associated with 14 wastewater sampling locations in Upstate New York were included in this analysis (Table 1). All samples were collected in September of 2020. A total of 28 samples did not have detectable SARS-CoV-2 RNA, one was detectable but not quantifiable, and 61 had quantifiable SARS-CoV-2 RNA. These results were detected during a time of relatively low SARS-CoV-2 transmission, with the wastewater catchment areas reporting less than five cases per day, or less than 15 cases per 100 000 population, on average (Table 1). All of the samples had quantifiable crAssphage DNA and 88 samples had quantifiable crAssphage cDNA. The remaining two samples were nondetectable for crAssphage cDNA.

The categorical comparison between the grab and composite samples showed 91.1% agreement (kappa *P*-value < .001) for detecting SARS-CoV-2 RNA (Table 2). There was no difference in the variance between grab and composite samples for the SARS-CoV-2 to crAssphage ratio (*P* = .23), the SARS-2-CoV RNA (*P* = .36), and crAssphage DNA (*P* = .43). Grab samples had lower variance than composite samples when assessing crAssphage cDNA (*P* = .04). The quantile–quantile plots also support that the grab and composite samples are similar, except for the crAssphage cDNA (Fig. 1). The correlations among the quantifiable grab and composite samples were statistically significant yet modest for SARS-CoV-2 RNA (Pearson correlation = 0.44, *P* = .02), crAssphage cDNA (Pearson correlation = 0.36, *P* = .02), and crAssphage DNA (Pearson correlation = 0.46, *P* = .002; Fig. 2). However, there was a wide range of variation in the correlation coefficients by county, with most not statistically significant, likely attributable to the small number of paired samples available for analysis in each county (Table 3).

**Table 2.** Detection agreement among grab and composite samples for SARS-2-Cov RNA, Upstate NY, 2020.

		Grab samples			Total
		Nondetectable	Detectable but nonquantifiable	Quantifiable	
**Composite samples**	Nondetectable	12	1	1	14
	Detectable but nonquantifiable	0	0	0	0
	Quantifiable	2	0	29	31
**Total**		14	1	30	45

**Table 3.** Correlations among quantifiable grab and composite samples, Upstate NY, 2020.

Location	SARS to CrAssphage ratio		SARS-2-CoV RNA		CrAssphage cDNA		CrAssphage DNA	
	Correlation	*P*-value	Correlation	*P*-value	Correlation	*P*-value	Correlation	*P*-value
Overall	0.51N = 29	.005	0.44n = 29	.02	0.36n = 43	.02	0.46n = 45	.002
Albany	0.40N = 14	.17	0.40N = 14	.15	0.31N = 30	.10	0.49N = 30	.007
Buffalo	0.89N = 8	.004	0.86N = 8	.006	0.63N = 8	.10	0.70N = 8	.05
Newburgh	−0.05N = 7	.91	−0.21N = 7	.66	0.82N = 5	.09	−0.16N = 7	.73

*Log values used in correlation analysis.

## Discussion

We found good comparison between grab and 24-hour composite samples for detecting SARS-CoV-2 RNA from municipal wastewater treatment plants as well as for crAssphage DNA. Our results suggest that grab samples may be sufficient to detect SARS-CoV-2 RNA in order to monitor presence/absence overtime. However, there is less agreement between the grab and composite samples when quantifying the amount of SARS-CoV-2 RNA in wastewater. Therefore, the appropriate sampling strategy depends on the goals of the surveillance program.

A comparison of samples collected from manholes during a period of high transmission in Iran found that grab and composite samples generally agreed for detecting SARS-CoV-2 RNA. However, grab samples reported less SARS-CoV-2 RNA in the wastewater than the composite samples (Rafiee et al. 2021). The large daily variations in the amount of human fecal matter at the subsewer shed level likely contributed to the poorer performance of the grab samples in this study (Rafiee et al. 2021). A group in Virginia found good agreement between grab and composite samples during a period of low transmission from samples collected at a municipal wastewater treatment facility (Curtis et al. 2020). However, neither of these studies examined the comparability of the types of sampling methods for detecting crAssphage DNA or other markers of human fecal matter.

This study was completed during a time of relatively low COVID incidence at municipal wastewater treatment plants. It is unclear how the grab samples would compare to the 24-hour composite samples during periods of more intense transmission. Our analysis demonstrates that grab and 24-hour composite samples perform equally well for detecting (or not detecting) SARS-CoV-2 RNA in municipal wastewater. Therefore, grab samples are an efficient and cost-effective method to monitor for the presence of SARS-CoV-2 in the entire community. This methodology can be used in place of autosamplers at locations where autosamplers are not feasible, in resource-limited settings, and when there are supply chain disruptions.

## Declaration of competing financial interests

H.G. holds a provisional patent on the UltraSucrose methodology (application number 63/039338). All other authors declare they have no actual or potential competing financial interests.

## Supplementary Material

xtac017_Supplemental_File
