# Supplementary material for: Comparability of 24-hour composite and grab samples for detection of SARS-2-CoV RNA in wastewater
Source: FEMS Microbes. 2022 Jun 17;3:xtac017. doi: 10.1093/femsmc/xtac017 (PMC10117866; doi:10.1093/femsmc/xtac017)
Supplement: xtac017_Supplemental_File [file xtac017_supplemental_file.docx]

**Reviews of manuscript FEMSMC-2021-074.r0**

**Reviewer 1**

**Recommendation: Major revision**

This manuscript by Kmush et al compares 24-hour composite samples with grab samples for detection of SARS-2-CoV RNA in wastewater. The manuscript is not particularly novel particularly considering there are multiple papers that already compare grab samples against composite samples. I think this type of study could benefit from a proper uncertainty assessment of “how many grab samples need to be taken to be XX% confident that there is infection within the community?”. For example, the authors should look at the works of Ort et al. (e.g. https://pubs.acs.org/doi/abs/10.1021/es100779n ) where assessments were made based on different scenarios of releases to sewer (pulses; in this case number of toilet flushes by shedding individuals), size of catchment population etc. Whether or not faecal DNA/RNA is sufficiently sampled using an autosampler should also be explored as there are many types of autosamplers, some with filters, some without, in high flowing channels there may be additional complications. Without doing this I fail to see what this study adds to the literature. Also, with the passive sampler work (Moore Swab – your reference 14), if lack of autosampler equipment is the limitation, it has already been shown that techniques such as Moore Swab can potentially even outperform an autosampler?

Other comments:

Introduction (Page 3/15)

Line 17-19: Many WWTPs already have autosamplers on the inlet and outlet of wastewater treatment plant. Additionally, small peristaltic pumps which can provide continuous sampling from a wastewater treatment plant can be readily bought from pump stores or even aquariums

Lines 21-26: Please have a look at the works of Ort et al where it’s clearly demonstrated that continuous flow proportional sampling is the gold standard.

Lines 30-37: Why not also look at passive sampling as an alternative?

Materials and Methods (Page 4/15)

Line 10: what is “well-timed”? Do you know the exact time people shedding flushed? Do you know the exact hydraulic retention times of the sewer catchments?

Line 19: What mode and frequency were the autosamplers working in? The argument that there’s a gold standard for this doesn’t hold up when it’s not described here.

Results (Page 6/15)

Lines 52-54: The important consideration here is the cases per catchment population eg, 5 cases in a catchment of 1000 people is very different to 5 cases in a catchment of a million people

Line 28: what’s the purpose of looking at correlation coefficients by county?

Discussion (Page 6/15)

Lines 37-39: Without considering the uncertainties and limitations this is an unfounded statement.

**Reviewer 2**

**Recommendation: Minor revision**

The MS reports comparative data of two sampling approaches for the application of WBE. Authors reports no significant difference between composite and grab sampling approach. This means application of WBE can be implement without expensive sampling equipment. Overall the MS is good however, conclusion can be condensed to the point.

**Author's Response to Decision Letter for (FEMSMC-2021-074)**

We thank the Reviewers for their insightful comments and feedback regarding our manuscript. Our detailed responses to your comments can be found below (bulleted).

Reviewer: 1

Comments to the Author

This manuscript by Kmush et al compares 24-hour composite samples with grab samples for detection of SARS-2-CoV RNA in wastewater. The manuscript is not particularly novel particularly considering there are multiple papers that already compare grab samples against composite samples. I think this type of study could benefit from a proper uncertainty assessment of “how many grab samples need to be taken to be XX% confident that there is infection within the community?”. For example, the authors should look at the works of Ort et al. (e.g. https://pubs.acs.org/doi/abs/10.1021/es100779n ) where assessments were made based on different scenarios of releases to sewer (pulses; in this case number of toilet flushes by shedding individuals), size of catchment population etc. Whether or not faecal DNA/RNA is sufficiently sampled using an autosampler should also be explored as there are many types of autosamplers, some with filters, some without, in high flowing channels there may be additional complications. Without doing this I fail to see what this study adds to the literature. Also, with the passive sampler work (Moore Swab – your reference 14), if lack of autosampler equipment is the limitation, it has already been shown that techniques such as Moore Swab can potentially even outperform an autosampler?

• We thank the reviewer for this comment and for suggesting the works of Ort et al. We have now incorporated that paper into our revised Introduction (end of the second paragraph).

• We respectfully disagree with the reviewer that this study does not add anything useful to the body literature. Our focus is on grab vs composite in sanitary sewer collection systems of a solids-associated RNA virus; there is not a broad range of literature available on topic. Given the comparison between autosampler results and grab sample results from this study, we can clearly see that although autosamplers are more sensitive to pick up evidence of a pathogen in wastewater, grab samples still work well. And the correlation between the levels is good. While passive samplers or Moore swabs might work better than grab samples, they are not without their own complications in use, particularly in terms of losing the swab. Grab samples are perhaps the simplest and easiest way to sample wastewater.

• A study that confirms the results of other published work is useful, if not essential. At the time of the development of this work, other sampling methodologies were considered, however; grab samples and composite samples represent the technologies most readily available and accepted for use in the field of environmental sampling. These sampling techniques may not always generate similar results. It is generally accepted that grab samples capture a single moment in time from a single sampling point. In a highly variable environment, such as is presented by the wastewater system, logic dictates that a grab sample may be inferior to a composite sample produced by an autosampler. While care can be taken to minimize some of the variability introduced by the use of a grab sample (e.g. sampler training, location chosen in relation to the wastewater system, timing of the sampling at one of the diurnal peaks typical for maximum fecal content, etc.) standard environmental sampling practice would be to achieve “signal averaging” though the use of a composite sample to reduce the potential for sample to sample variability. All of the above is especially true when trying to monitor for a pathogen during periods of low transmission as was the case when this study was performed.

• Unfortunately, we did not conduct enough experiments to examine how case loads, sewershed size, etc. affect the sensitivity and comparability of grab samples to composite samples. That is precisely why this study needs to be published, so that in a future systematic review and meta-analysis these results can be combined with other similar studies to gain sufficient statistical power to make these types of comparisons. Ort et al. included 87 studies in their review – how would they find those studies if manuscripts such as ours were rejected for not being “particularly novel”?

Other comments:

Introduction (Page 3/15)

Line 15-17: Many WWTPs already have autosamplers on the inlet and outlet of wastewater treatment plant. Additionally, small peristaltic pumps which can provide continuous sampling from a wastewater treatment plant can be readily bought from pump stores or even aquariums

• We agree with the reviewer on this comment. Most, but not all, municipal/utility wastewater treatment plants have autosamplers on the influent and discharged effluent from the facility. There is sample volume and timing control on these commercially available pieces of equipment. This can be flow proportioned sampling, as often there is flow measurement at one or both locations. Small peristaltic pumps are readily available. However, the goal of this study was to compare existing and industry accepted sampling equipment, not to create novel or cheap sampling equipment.

Lines 21-26: Please have a look at the works of Ort et al where it’s clearly demonstrated that continuous flow proportional sampling is the gold standard.

• We agree with the reviewer, we have changed the language in the Introduction (beginning of the first paragraph). However, since we preformed time-proportional sampling our study, we have left in the language describing time-proportion collection. The decision to perform time proportional sampling was due to the added complexity presented by flow proportional sampling. Wastewater flow can be challenging to measure and compensate for in an actual wastewater system at the points that are readily available for access and sampler installation. Furthermore, in cases where mains receive large non-residential inputs, it may be possible for flow proportional sampling to negatively impact the sample viability - e.g. when sampling downstream of a factory that releases a chemical that may interfere or degrade the sample.

Lines 30-37: Why not also look at passive sampling as an alternative?

• The goal of this study was to evaluate grab samples and composite samples. Other sampling strategies were not part of the study.

Materials and Methods (Page 4/15)

Line 10: what is “well-timed”? Do you know the exact time people shedding flushed? Do you know the exact hydraulic retention times of the sewer catchments?

• Yes, we did know the hydraulic retention times of the specific sewer catchments and the grab samples were timed during the morning diurnal peak. This clarification has been added to the Methods (Study Design paragraph).

Line 19: What mode and frequency were the autosamplers working in? The argument that there’s a gold standard for this doesn’t hold up when it’s not described here.

• We apologize for leaving this information out. Autosamplers were set to collect at a frequency and sampling volume within parameters set forth by the US Environmental Protection Agency, this has been added to the Methods (Grab and 24-hour Composite Sample Collection 1st paragraph).

Results (Page 6/15)

Lines 52-54: The important consideration here is the cases per catchment population eg, 5 cases in a catchment of 1000 people is very different to 5 cases in a catchment of a million people

• We have edited the text to reflect the absolute number of cases as well as the per 100,000 population number of cases (1st paragraph of the results). Table 1 has also been updated to include a new column.

Line 28: what’s the purpose of looking at correlation coefficients by county?

• Correlation analysis assumes that all data points are independent of each other. Due to the hierarchical nature of the data (multiple treatment plants within the same country), this assumption was not met. However, as the sample size gets smaller by subdividing that data into each level, we lose statistical power. Therefore, we examined the correlations overall and within county, the former to keep statistical power and the later to meet the independence assumption.

Discussion (Page 6/15)

Lines 37-39: Without considering the uncertainties and limitations this is an unfounded statement.

• We thank the reviewer for this comment. We have changed the language to reflect the uncertainty.

Reviewer: 2

Comments to the Author

The MS reports comparative data of two sampling approaches for the application of WBE. Authors reports no significant difference between composite and grab sampling approach. This means application of WBE can be implement without expensive sampling equipment. Overall the MS is good however, conclusion can be condensed to the point.

• We thank the reviewer for this feedback. It is important to have peer-reviewed evidence of the utility of different types of sampling strategies-a simple, but useful conclusion.
